# The Combined Effect of Corn Zein and Buckwheat Flour on the Rheological, Microstructural, Physicochemical, and Nutritional Properties of Gluten-Free Bread

**DOI:** 10.3390/foods15173018

**Published:** 2026-08-27

**Authors:** Gaukhar Akshorayeva, Gulnazym Ospankulova, Vural Gökmen, Svetlana Kamanova, Linara Murat, Bakhyt Shaimenova, Daulet Aitmukhanbetov, Sayagul Tazhina, Mukhtarbek Kakimov

**Affiliations:** 1 The Department of Food Technology and Processing Products, Saken Seifullin Kazakh Agrotechnical University, Astana 010000, Kazakhstan; gaukharmadok@gmail.com (G.A.); bulashevag@mail.ru (G.O.); kamanovasveta@mail.ru (S.K.); linaraazamatkyzy@mail.ru (L.M.); bshaymenova@mail.ru (B.S.); kerei@mail.ru (D.A.); sayagultazhina@gmail.com (S.T.); 2 Food Quality and Safety (FoQuS) Research Group, Department of Food Engineering, Hacettepe University, Beytepe, 06800 Ankara, Türkiye

**Keywords:** buckwheat, corn zein, gluten-free bread, protein–starch interactions, dough rheology, bread quality

## Abstract

The development of structurally stable and nutritionally enhanced gluten-free bread is a major technological challenge due to the absence of a gluten network. This study investigates the role of zein, a hydrophobic corn prolamin, in green buckwheat dough systems and its impact on bread properties. Zein was extracted from corn gluten and incorporated at 0, 10, 20, and 30%. Zein incorporation significantly modified starch–protein interactions, promoting a composite matrix with enhanced thermomechanical stability. The 20% zein formulation showed optimal performance, with increased peak viscosity, higher C2 and C3 torque values, improved starch gelatinization, and a more cohesive microstructure. The scanning electron microscopy analysis confirmed a continuous protein–starch network, with the highest elasticity and cohesiveness (0.85 and 0.57, respectively). The optimized formulation maintained functional value and reduced the conditional glycemic index. In the 20% zein formulated sample, the rapidly digestible starch fraction decreased from 48.1% to 41.6%, while slowly digestible and resistant starch increased from 22.4% and 7.8% to 26.3% and 10.4%, respectively. Overall, zein enrichment effectively compensates for the absence of gluten, resulting in improved bread quality and controlled starch digestibility. These findings provide new insights into protein–starch interactions in gluten-free systems and support the application of zein as a functional structuring agent in pseudocereal-based bakery products.

## 1. Introduction

Currently, the production of gluten-free bread products is one of the most relevant areas in food technology. The absence of gluten complicates the formation of an elastic structure in the dough and negatively affects the quality of the finished product (volume, porosity, softness). Therefore, the use of structuring agents in gluten-free systems and the scientific justification of their dosage is an important task.

The development of bread based on green buckwheat using zein as a structuring protein is at the intersection of two rapidly developing areas: gluten-free products and functional bakery products. Green buckwheat (*Fagopyrum esculentum*) attracts attention due to its high content of protein, dietary fiber, antioxidants (rutin, phenolic acids), and absence of gluten, as well as its favorable amino acid profile. Reviews on buckwheat in gluten-free products show that buckwheat flour improves the structure and nutritional value of bread compared to many other gluten-free cereals and pseudocereals [1].

Zein, a hydrophobic prolamin protein prevalent in corn, emerges as a promising functional gluten substitute. It possesses the ability to form an elastic, film-forming matrix, thereby partially mitigating the absence of gluten in gluten-free dough [2].

In systems based on green buckwheat, where the natural protein fraction does not form a continuous gluten network, issues of zein dosage and functional state become key to dough rheology and the quality of the finished bread. Although Li et al. [3] investigated the impact of incorporating corn prolamin on the properties of buckwheat dough, their research primarily focused on assessing the structural and rheological characteristics of the dough. The influence on the dough’s texture was not considered. Buckwheat flour is characterized by a high content of starch (about 60–70%), protein (10–15%), dietary fiber, and minerals; at the same time, proteins are mainly represented by globulins and albumins, rather than prolamins, as in wheat. This results in a weak ability to form an elastic network but contributes to increased viscoelasticity due to starch and fiber. Studies devoted to various fractions of buckwheat flour (by particle size, degree of processing, germination) show that its technological properties strongly depend on the structure of starch, the content of bran fraction, and enzyme activity. Thus, medium-sized fractions provide an optimal combination of water absorption capacity, gas retention capacity of the dough, and bread quality (volume, porosity, texture) [4].

High enzymatic activity and the presence of heat-sensitive antioxidants are especially important for green (unroasted) buckwheat. Studies on sprouted and fermented buckwheat show that such treatments alter the gelatinization and pasting properties of starch, shift the gelatinization temperature, and increase the elasticity of the dough [5]. This creates the conditions for the formation of a more stable crumb structure, if hydration and kneading time are correctly adjusted. Nevertheless, most of the studies mentioned above examine the impact of green buckwheat in systems that do not consider the interaction with additional protein components, such as zein. There is a lack of data on how the behavior of starch and non-protein components of green buckwheat changes in the presence of hydrophobic prolamin protein and how this affects the early stages of dough formation [6]. Zhang Y. et al. (2021) provided an overview of zein in gluten-free systems, and the article points to the need for further research [7]. Reviews on zein in gluten-free products show that this prolamin protein, due to its hydrophobic nature and ability to self-assemble, forms continuous, albeit relatively fragile, networks in starchy matrices [8]. A systematic review of gluten-free recipes shows that zein is one of the functional proteins (along with milk and soy) that increase specific volume and reduce crumb hardness; however, sensory characteristics are not always improved due to the specific taste and color of zein [9]. At the same time, a review of the literature emphasizes that these factors have been studied only fragmentarily to date, and the combination of green buckwheat and zein has hardly been studied at the level of a systematic approach [10].

Several studies have investigated the extraction, structural characteristics, and functional properties of zein obtained from corn gluten meal (CGM), corn fermentation coproducts, and other industrial by-products These studies have predominantly focused on zein recovery, structural characterization, film formation, adhesive applications, or functionality in model systems, with limited evidence regarding the performance of purified zein in gluten-free bakery products. Paraman and Lamsal (2011) recovered and characterized α-zein from corn ethanol coproducts and demonstrated its purity and film-forming properties; however, its functionality in gluten-free bread was not investigated [11]. Zheng et al. (2014) demonstrated that extrusion and starch-removal pretreatments affected the structural characteristics of zein extracted from CGM, but the functional performance of the resulting zein in a real food matrix was not evaluated [12]. Ozturk and Mert (2018) investigated the effects of microfluidization of CGM on the rheological and textural properties of gluten-free corn bread; however, the study used microfluidized CGM rather than purified zein and did not address the role of zein in starch digestibility [13]. Peng et al. (2024) further demonstrated that the extraction procedure and source characteristics can influence the structural and functional behavior of zein in composite gel systems, highlighting the importance of zein processing and purification for its subsequent functionality [14]. Sanvezzo et al. (2026) developed a sustainable extraction approach for zein from CGM for adhesive applications, further demonstrating the potential of CGM as a valuable secondary raw material, but outside the food-bakery context [15].

The present study addresses an important research gap by investigating purified zein derived from corn gluten meal as a functional protein ingredient in gluten-free bread based on buckwheat flour. Zein was incorporated at three concentration levels—10, 20, and 30%, enabling systematic assessment of its concentration-dependent effects on dough rheology, bread texture, microstructure, physicochemical characteristics, color, and nutritional properties. In contrast to previous studies that primarily examined zein extraction and characterization or its functionality in non-bakery and model systems, the present work evaluates the behavior of CGM-derived zein directly within a gluten-free bread matrix and examines its contribution to overall product quality. An additional feature of the formulation was the inclusion of corn syrup at a constant level in all bread formulations. Rather than serving as an independent experimental factor, the corn syrup was incorporated uniformly across all treatments to maintain comparable formulation and fermentation conditions and to ensure that the observed differences among samples could be primarily attributed to the level of zein incorporation. This experimental design provides a controlled basis for elucidating the functional role of zein in a buckwheat-based gluten-free bread system. Overall, the novelty of this study lies in the integrated evaluation of CGM-derived zein as a functional structuring protein in buckwheat-based gluten-free bread, linking its concentration-dependent effects on dough behavior and bread quality with microstructural, textural, physicochemical, and nutritional characteristics. This approach extends the current understanding of zein functionality from extraction and model-system studies toward its practical application as a value-added ingredient in gluten-free bakery products.

## 2. Materials and Methods

### 2.1. Materials and Chemical Reagents

Flour from unprocessed buckwheat (protein 12.6 g, fat 3.3 g, carbohydrates 57 g, fiber 1.3 g) was obtained from Kazyna LLP (Astana, Kazakhstan). Corn gluten (protein 65.9%, fat 35.8%, moisture 8%) was obtained from AsiaAgroFood LLP (Almaty, Kazakhstan). Enzymatic corn syrup was purchased at a local supermarket manufactured by the Zharkent Starch and Syrup Plant LLP (Almaty, Kazakhstan). Ethanol, petroleum ether, and other analytical grade reagents were purchased from Sigma-Aldrich (St. Louis, MO, USA). All experiments were conducted in triplicate.

### 2.2. Extraction of Zein from Corn Gluten

Zein was extracted and purified from corn gluten meal according to the method of Takahashi and Yanai (1996) [16], with modifications. A 500 mL portion of n-hexane was added to 100 g of corn gluten meal, and the mixture was homogenized and then subjected to 1 h of reflux extraction in a boiling water bath. After cooling, hexane solution was separated by filtration. This hexane solution contained 3.9 g of oily solid substances having a slightly yellow color. Next, 800 mL of 95% ethanol was added to the remaining corn gluten meal and mixed for 15 min in a homogenizer arranged in a water bath of 70 °C. The resulting mixture was centrifuged at 2500 rpm for 2 min at the same temperature to obtain a light yellow and transparent zein extract.

The thus-obtained extract was cooled to −10 °C. to cause precipitation of zein and then subjected to solid–liquid separation by decantation to obtain 44.6 g of a precipitate. When 85% ethanol was added to this precipitate to a total weight of 100 g, a clear zein solution (solid content, 20.0%) was obtained.

The precipitate was subjected to lyophilization at a residual moisture content corresponding to approximately 20% dry matter, using a laboratory freeze dryer (Telstar Cryodos, Telstar, Terrassa, Spain) at a chamber pressure of 0.150 ± 0.010 mbar. The resulting material was considered as a model sample for further study of its rheological and physicochemical characteristics.

The purity of corn zein was determined using the Kjeldahl method according to the standardized procedure described by Thiex et al. (2002) [17], which corresponds to the AOAC Official Method 2001.11 for the determination of nitrogen in grain crops and protein-containing materials. A 1 g sample was mineralized with concentrated sulfuric acid with a catalyst, converting organic nitrogen into ammonium salts. Further alkaline decomposition released ammonia, which was distilled off and titrated with acid. The protein content was calculated using the conversion factor N × 6.25.

### 2.3. Determination Physicochemical Properties of Buckwheat Flour and Corn Zein

Moisture content was determined in accordance with the official method AACC 44-15.02.2010 [18], widely used for grain products and protein powders. The method is based on drying the sample to a constant weight, which ensures high reproducibility for materials containing both free and weakly bound moisture.

The fat content was determined according to AOAC 920.85 using classical extraction in a Soxhlet apparatus. A 6 g sample was extracted with petroleum ether in a SOXTHERM SOX414 system (Gerhardt, Königswinter, Germany) for 4 h.

The ash content was determined using [19], which involves calcining a 2 g sample in a porcelain crucible at 600 °C for 6 h in a muffle furnace (Snol 7.2/1100, No10752, Snoltherm, UAB, Utena, Lithuania).

The starch content was determined according to a method modified from Feng Kong et al. [5]. An amount of 50 mg of flour was mixed with 6 mL of KOH (2 M) for alkaline hydrolysis, loosening its structure. Next, the mixture was incubated at room temperature for 30 min. An amount of 3 mL of sodium acetate (0.4 M, pH 4.75) was added, and pH was adjusted to 4.75 with HCl solution (1 M). Then, amyloglucosidase was added (60 µL; 1.67 × 10^−4^ kat/mL) and incubated at 60 °C for 45 min. Determination of glucose and calculation of starch content was done by a factor of ×0.9.

### 2.4. Preparation of Buckwheat–Zein Doughs and Breads

The Dough preparation process was divided into stages: (i) heating the glucose syrup, zein and water mixture to 50–60 °C to ensure a lower viscosity; (ii) cooling the liquid mixture to 28–35 °C, a temperature safe for yeast; and (iii) mixing the dry components with the liquid mixture, and yeast was added at this stage. The technological solution was based on separating the temperature and time regimes for the liquid and dry phases: the liquid phase was heated to 50–60 °C to reduce viscosity, then cooled to 28–35 °C before adding yeast and mixing with the dry phase. This prevented the thermal denaturation of zein and flour proteins during the mixing stage, preserving their functional properties while ensuring yeast activity.

The bread samples were prepared using the AACC International Method 10-10.03, with some modifications. The base recipe included sourdough starter, buckwheat flour, fresh yeast (1.5 g/100 g), salt (1 g/100 g), vegetable oil (10 g/100 g), and glucose syrup (10 g/100 g). Zein was mixed with buckwheat flour in ratios of 0, 10, 20, and 30%, and the dough was coded as BZ0, BZ10, BZ20, and BZ30, respectively. The buckwheat flour, zein and salt were thoroughly mixed. The glucose syrup and fresh yeast were dissolved in a small amount of warm water. The main portion of the water was heated to 55 °C before being added to the dry mixture. The mixture was stirred for 5–8 min at high speed. For the starter, fresh yeast was mixed with room-temperature water, a portion of the flour was added, and the mixture was placed in a fermentation cabinet (EMP.MF4–6, Empero, Konya, Türkiye) for fermentation. The ingredients were then mixed in a planetary mixer (5K45SSBWH, KitchenAid, Greenville, OH, USA) for 10–12 min. Subsequent fermentation was carried out at a temperature of 35–38 °C and a relative humidity of 85% for 70 min. The breads were baked at a temperature of 210 °C, reducing the temperature to 190 °C for 30 min in an electric rotary oven (ZU-BABY60X80E, Tecnoservice’21, Fano, Italy).

### 2.5. Determination of Dough Quality Parameters

Pasting behavior: The pasting behavior of the dough samples was evaluated using a Rapid Visco Analyzer (RVA 4500, Perten Instruments, Hägersten, Sweden) according to a standard temperature profile widely used in studies of starch-containing products. The suspensions were heated from 50 to 95 °C at a constant rate, and the viscosity was recorded at six temperature points (50, 60, 70, 80, 90, and 95 °C). This approach allows for the evaluation of key thermomechanical transitions, including initial swelling, gelatinization, granule destruction, and high-temperature stability. The method is particularly relevant for gluten-free systems, in which protein–starch interactions significantly influence pasting behavior [20].

The test measures changes in viscosity during controlled heating and cooling, reflecting starch gelatinization, α-amylase activity, and retrogradation:

(1) $$M_{2}\;=\;((100\;-\;14)\;\times \;M_{1})/(100\;-\;W_{2})$$

(2) $$W_{2}=25.0+(M_{1}-M_{2})$$

where *M*_1_ is the initial weight of the sample (3.50 g), *W*_1_ is the actual moisture content (%), *M*_2_ is the adjusted weight, and *W*_2_ is the adjusted water volume (mL). Key parameters were extracted from the viscosity curve to assess flour quality.

Thermomechanical behavior: The thermomechanical behavior of the dough samples was evaluated using a Mixolab 2 (Chopin Technologies, Villeneuve-la-Garenne, France) operated under the Chopin+ protocol. This method allows simultaneous monitoring of dough development and its response to mechanical shear and thermal treatment, providing integrated information on protein weakening, starch gelatinization, enzyme activity and starch retrogradation. Doughs were prepared according to the optimized water absorption for each formulation and immediately subjected to Mixolab testing. The measurement was conducted at a constant mixing speed using the standard Mixolab bowl and Z-shaped kneading arms [21].

After the initial mixing period, the dough was heated from 30 to 90 °C at a rate of 4 °C/min. This heating phase induced progressive protein denaturation and starch gelatinization. In the early part of the temperature ramp, the decrease in torque (from C1 to C2) was associated with protein weakening, i.e., the loss of mechanical strength of the protein network under combined thermal and mechanical stress. At higher temperatures, the torque increased again (C3), reflecting starch gelatinization, swelling of starch granules and the development of a continuous pasting matrix. The magnitude and position of the C3 maximum provided insight into the gelatinization behavior of starch in the buckwheat–zein system, as well as the impact of formulation on the ability of the starch phase to build viscosity under heating.

The dough was then held at 90 °C for 7 min, corresponding to a high-temperature holding phase. During this stage, the partial decrease in torque (C3→C4) was used to assess the thermal stability of the starch paste and the activity of amylolytic enzymes, if present. A steeper drop indicates higher enzyme activity and/or weaker paste stability, whereas a more stable plateau suggests heat-resistant starch structures and limited enzymatic degradation. For formulations based on buckwheat flour and zein, this segment of the curve is particularly informative, as it reflects the interactions between gelatinized starch, non-starch polysaccharides and added hydrophobic protein.

Finally, the sample was cooled from 90 back to 30 °C at the same rate (4 °C/min). In this cooling phase, the increase in torque (C5) was interpreted as a measure of starch retrogradation and network re-structuring during temperature decrease. The extent of torque recovery and the final C5 value provided information on the tendency of the starch phase to recrystallize and form a firmer gel, which is directly related to crumb firming and staling behavior during storage. Lower C5 values are usually associated with reduced retrogradation and potentially slower staling, whereas higher values indicate a stronger tendency toward re-association of amylose and amylopectin chains.

Microstructural analysis: The microstructure of dough samples was examined using scanning electron microscopy (SEM) to visualize the effect of zein addition on the organization of the starch–protein matrix. Dough samples containing 0, 10, 20 and 30% zein (based on flour weight) were prepared according to the optimized formulations and processed following a protocol adapted from previous studies on gluten-free and zein-based doughs [22]. SEM observations were performed using a Hitachi S-4800 field-emission scanning electron microscope (Hitachi, Tokyo, Japan). The microstructural images were acquired using an in-lens detector at an accelerating voltage of 5.0 kV, a working distance of 4.0–5.7 mm, and a probe current of 100 pA.

### 2.6. Determination of Bread Quality Parameters

Specific Volume: After cooling for 2 h, the loaves were measured for volume using the rapeseed displacement method. The mass of the samples was determined on analytical scales. The specific volume was calculated as the ratio of volume to mass (mL/g).

Baking Loss: Losses were determined by the difference between the mass of the dough before baking and the mass of the bread after cooling:

(3) $$Baking\;Loss,\;\%=\frac{m_{0}-m_{1}}{m_{0}}\times 100\;$$

where $m_{0}$ is the mass of the dough and m_1_ is the mass of the finished bread.

Moisture: Samples (5 g) were dried at 105 °C to a constant mass. Moisture content was expressed as a percentage of water by mass.

Color: The L*, a*, and b* color parameters were measured using a benchtop spectrophotometer (YS6010, Shenzhen 3NH Technology Co., Ltd., Shenzhen, China). Measurements were taken on the crust and in the center of the crumb, in three repetitions.

Texture profile analysis (TPA) was performed on the central part of the crumb, which is generally considered more homogeneous and less affected by crust-related artifacts [23]. The loaves were sliced and crumb cubes (2 × 2 × 2 cm^3^) were carefully cut from the center of each slice, avoiding the crust and peripheral regions. Texture measurements were performed using a texture analyzer (ST-2, Laboratory of Quality LLC, Moscow, Russia).

The primary TPA parameters—hardness, springiness and cohesiveness—were extracted from the force-time curves using the exponent software. Hardness was defined as the maximum force recorded during the first compression cycle and represents the resistance of the crumb structure to deformation. Springiness was calculated as the ratio between the sample height recovered after the first compression and the original height, derived from the time interval between the end of the first and the start of the second compression. Cohesiveness was expressed as the ratio of the area under the curve of the second compression to that of the first compression, reflecting the internal structural integrity and the ability of the crumb matrix to withstand repeated deformation.

In vitro digestion of starch and calculation of the conditional glycemic index: In vitro starch digestion was performed according to the method of Goñi et al. (1997) [24] with slight modifications. Simulated gastric digestion was performed in two stages: gastric digestion phase: a sample of crumb (0.5 g) was suspended in 0.1 M HCl, pepsin (40 mg/mL) was added, and the mixture was incubated for 30 min at 37 °C with shaking. Intestinal digestion phase: the pH was adjusted to 6.9 and α-amylase and pancreatic amylase were added. Samples were taken after 0, 20, 60, 120, and 180 min. The amount of glucose released was determined using the glucose oxidase method [25].

Sensory evaluation: Sensory evaluation was conducted after consideration and approval of the study protocol in accordance with the internal regulations of Saken Seifullin Kazakh Agrotechnical Research University. The sensory assessment protocol was developed with consideration of the general principles of sensory profiling described in ISO 13299:2016 [26]. The procedure included the selection of relevant sensory attributes, use of a standardized rating scale, preliminary familiarization of the assessors with the evaluation procedure, controlled presentation of coded samples, independent scoring, and subsequent processing of the obtained data. The sensory evaluation involved eight adult volunteer assessors. Participation was voluntary, and informed consent was obtained from all participants prior to the assessment. The procedure was non-invasive and non-interventional and did not involve the collection of sensitive personal or health-related information. Individuals with known allergies or intolerance to any ingredients used in the tested bread samples were excluded from the sensory evaluation. Four gluten-free bread formulations were evaluated: BZ0-0% zein, BZ10-10% zein, BZ20-20% zein, and BZ30-30% zein. The bread samples were prepared in the laboratory on the day of the sensory evaluation. After cooling, the crust was removed and each loaf was cut into eight approximately equal pieces. The samples were assigned random three-digit codes to minimize potential bias associated with sample identification. Each assessor received one coded portion of each formulation together with an individual evaluation sheet and drinking water for palate cleansing between samples.

A structured 9-point scale was used to evaluate the sensory characteristics of the bread samples. The evaluated attributes included appearance, crumb color, aroma, taste, texture, and overall acceptability. For the sensory attributes, a score of 1 corresponded to very low or barely perceptible expression of the attribute, whereas a score of 9 indicated a very high degree of expression. Overall acceptability was evaluated as an integrated assessment of the sensory quality of each bread sample using the same 9-point scale. The samples were evaluated independently, and assessors were instructed to cleanse their palate with water between samples before proceeding to the next formulation. The order of sample presentation was controlled to minimize the potential influence of the preceding sample on subsequent evaluations. Each assessor independently assigned a score to each sensory attribute for all four formulations. Following completion of the sensory evaluation, the individual scores were compiled and analyzed. For each sensory attribute and bread formulation, the mean score and standard deviation (SD) were calculated based on the evaluations provided by the eight assessors. The resulting values were used to characterize and compare the sensory profiles of the gluten-free bread formulations and to determine the formulation demonstrating the most favorable overall sensory characteristics.

### 2.7. Statistical Analyses

The analysis of variance (ANOVA) of the results was performed by using SPSS statistical program version 27 (Chicago, IL, USA), and Tukey multiple comparisons test was employed to evaluate the differences between groups with a 95% confidence interval.

## 3. Results

### 3.1. Physical and Chemical Quality Indicators

The physicochemical composition of buckwheat flour and corn zein (Table 1) demonstrates their complementary technological functions in gluten-free bread formulations. Buckwheat flour contained 12.8% moisture, 11.28% protein, 2.23% fat, 2.67% ash, and 71.02% carbohydrates, values consistent with those reported for whole-grain buckwheat flour in recent studies. The relatively high ash content reflects the presence of mineral-rich outer grain layers, while the predominance of starch provides the structural basis for gelatinization and crumb formation during baking. In contrast, corn zein exhibited 89.56% protein, 8.0% moisture, and 0.95% ash, confirming its high purity and concentration as a storage protein isolated from maize endosperm [7].

The substantial compositional differences between buckwheat flour and zein explain their distinct technological roles during dough development. The starch-rich buckwheat flour governs water absorption, viscosity development, and gelatinization, whereas the highly hydrophobic zein contributes primarily to the formation of a continuous protein network after thermal treatment. Since zein exhibits limited hydration under ambient conditions, heating the liquid phase containing glucose syrup to 50–60 °C, followed by cooling to 28–35 °C before yeast addition, promoted more uniform protein dispersion while preserving yeast viability. Such a processing strategy facilitates protein–starch interactions, improves gas retention, and limits excessive starch swelling and amylose leaching during baking [7].

The incorporation of glucose syrup further contributed to dough functionality by reducing the viscosity of the liquid phase during heating, improving the hydration of both starch and zein, and serving as a fermentable substrate for yeast. In addition, glucose syrup acts as a humectant, enhancing moisture retention and slowing crumb firming during storage. Therefore, the combination of starch-rich buckwheat flour and protein-rich zein, together with the optimized thermal treatment of the glucose syrup solution, created a balanced gluten-free system capable of improving dough stability, loaf volume, and crumb softness and delaying starch retrogradation compared with conventional gluten-free formulations [7].

### 3.2. Pasting and Thermomechanical Behavior of Doughs

The pasting properties (Table 2) demonstrated that corn zein significantly affected the thermal behavior of the buckwheat starch system. Peak viscosity increased significantly (*p* < 0.05) with increasing zein concentration, rising from 1105 cP for BZ0 to 2017 cP for BZ30, indicating progressive strengthening of the starch–protein matrix and improved resistance of starch granules to thermal and mechanical disruption. Similarly, trough and final viscosities reached their highest values in BZ30, confirming enhanced paste stability during heating and cooling. In contrast, the pasting temperature showed a different trend, increasing at 10% zein but decreasing significantly to 72.6 °C at 30% zein, suggesting that the combined effect of thermally hydrated zein and glucose syrup facilitated starch gelatinization. These findings indicate that zein modifies starch pasting behavior by reinforcing the continuous matrix surrounding swollen starch granules, thereby improving paste stability during thermal processing.

Coţovanu et al. (2022) showed that the optimal proportion of buckwheat flour and its average particle size provide a compromise between dough viscosity and extensibility: an excessively fine fraction leads to excessive water absorption and compaction of the structure, while a coarse fraction leads to uneven porosity and a reduction in bread volume [27]. The Chopin+ protocol was used to evaluate water absorption capacity, dough formation and stability parameters, as well as key torque values C2, C3, and the difference between C5 and C4, traditionally interpreted as indicators of protein thermal weakening, starch gelatinization, and starch retrogradation, respectively. The thermal torque parameters C2 and C3 reached their maximum values in BZ20, demonstrating significantly greater protein resistance to thermal weakening and enhanced starch gelatinization compared with the control. No significant difference in C2 was observed between BZ10 and BZ30, suggesting that further zein addition beyond 20% did not improve protein thermal stability. Likewise, although BZ20 exhibited the highest C5−C4 value, it was statistically comparable with BZ30, indicating that increasing zein from 20% to 30% did not further enhance starch reassociation during cooling. Overall, these results suggest that 20% zein provided the optimum balance between dough development, protein stability, and starch gelatinization, whereas 30% zein mainly increased paste viscosity without producing proportional improvements in the rheological characteristics of the dough. Water absorption increased significantly after zein incorporation, whereas no significant differences were observed among the zein-containing formulations. Dough development time differed significantly among all samples, reaching its maximum in BZ20 and decreasing in BZ30, suggesting that 20% zein favored progressive protein–starch network formation, while 30% zein accelerated dough structure development and reduced the time required to reach optimum consistency. Despite this shorter development time, dough stability remained statistically comparable among BZ0, BZ20, and BZ30, indicating that higher zein incorporation did not impair the mechanical resistance of the dough during mixing.

### 3.3. The Structure-Forming Role of Zein in Gluten-Free Systems

At the micro level, it has been shown that in composite doughs, zein can form thin fibrillar structures that partially envelop the gluten network or starch granules, increasing the resistance of gas cell membranes to rupture. This structural function is especially important for systems lacking natural gluten, such as buckwheat dough.

**Figure 1:**
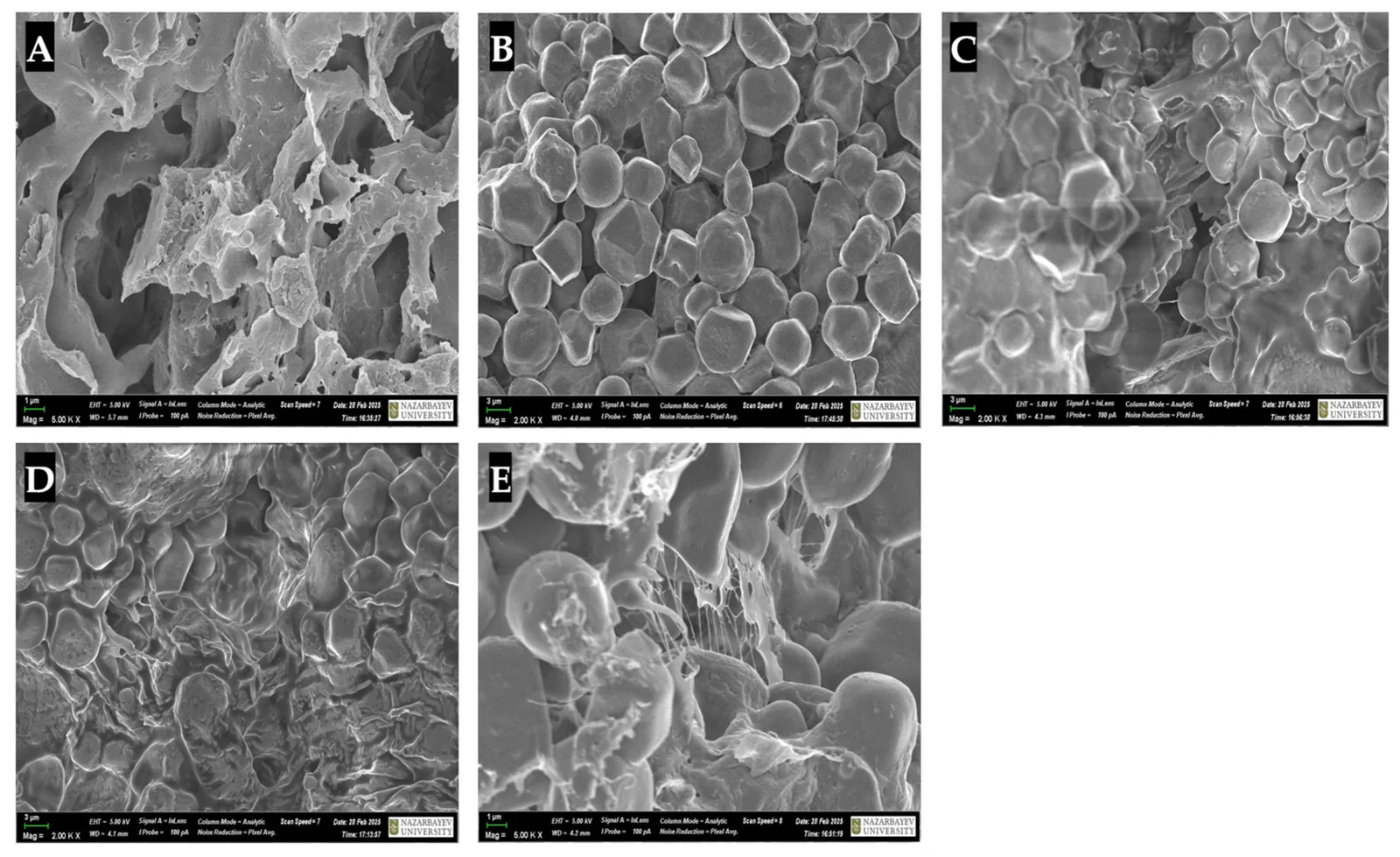
SEM images of corn zein and buckwheat + zein based dough samples: (**A**) microstructure of zein; (**B**) no zein; (**C**) 10% zein; (**D**) 20% zein; (**E**) 30% zein.

The SEM images (Figure 1) demonstrate the characteristic fibrous and lamellar microstructure of zein, consisting of elongated thread-like formations and densely ordered lamellar domains. This morphology is due to the aggregation characteristics of α-zein, a hydrophobic prolamin protein that tends to form long fibrils and film structures during drying. The images clearly show long parallel fibers, as well as areas of their cross-linking, reflecting a high degree of internal structural organization. Similar fibrosity of zein has previously been noted in studies of its film-like properties and nanostructuring [28], confirming its ability to act as a natural biopolymer reinforcing component in composite matrices.

Morphological changes recorded using SEM demonstrate the fundamental influence of zein on the structural organization of green buckwheat dough and provide a deeper understanding of the mechanisms underlying the rheological results of Mixolab and RVA. The control sample, which does not contain zein, is characterized by a structure typical of buckwheat flour rich in pseudo-grain starch granules: the granules are predominantly separated from each other, their surfaces are smooth, and the intergranular spaces are pronounced. This morphology corresponds to weak matrix integration and explains the low C2 and C3 values, since the absence of a protein network limits the ability of the dough to maintain mechanical stability and form a structured gel when heated. Similar observations have been noted in the works of Sciarini et al. (2010) [29], where gluten-free systems demonstrate low cohesion and high granularity. BZ10 shows a pronounced tendency to form primary protein–starch complexes.

The SEM images show partial coverage of the granules with thin fibrillar and film-like structures characteristic of α-zein, known for its ability to form nanofibrils and lamellar films. This level of addition leads to local strengthening of the matrix, which manifests itself in an increase in water absorption and dough development time. However, the structure remains heterogeneous, which is consistent with the moderate growth of C2 and C3 recorded on Mixolab. These results are reminiscent of those reported by Lai H.M. and Padua G.W. [30], who reported that low doses of zein create only partial interactions, without providing complete structural rearrangement of the gluten-free matrix.

The maximum morphological effect is achieved with BZ20 (photo D). The SEM images show a dense, cohesive, composite structure in which starch granules are embedded in a protein matrix, and the boundaries between them are partially blurred and stabilized. In this case, zein functions as a structuring agent like gluten in traditional doughs: its hydrophobic domains form a fibrous network that binds the granules together. This concentration level provides an optimal balance between starch swelling and structure retention, as confirmed by the peak values of C2 and C3, as well as improved thermal stability (increased C5−C4). Similar structural optimization in composite systems was also noted in studies by Sadat et al. [31], demonstrating that zein forms fibrillar protein networks capable of stabilizing starch-based dough systems and improving structural integrity. BZ30 (E) leads to a noticeable change in morphology: the graininess of the structure decreases, but an excessive amount of protein phase appears, forming thick surface coatings and agglomerates.

The SEM images show signs of protein network over compaction, as well as uneven zein distribution. This morphology naturally explains the decrease in peak rheological properties: an excessive amount of protein limits starch hydration, leads to a decrease in gelatinization, and prevents the formation of optimal starch–protein bonds. Similar effects of protein overload have been described by Skendy et al. [32], where high levels of prolamins impaired plasticity and reduced the dough’s ability to swell and retain gas.

### 3.4. Quality Characteristics of Breads

Baking loss decreased significantly from 11.8% in the control to 10.1% in BZ20, while BZ30 showed an intermediate response and did not differ significantly from either the control or BZ10. Likewise, crumb moisture increased from 38.4% in the control to 40.3–41.1% in zein-containing breads, although no significant differences were observed between BZ10 and BZ20, and BZ30 remained statistically comparable with both groups. These results indicate that 20% zein provided the optimum balance between structural reinforcement and water retention. Although the concentration of glucose syrup remained constant in all formulations, its hygroscopic properties likely contributed to moisture retention within the crumb, reduced water migration during baking, and delayed crumb firming during storage. Therefore, the combined action of zein as a structural protein and glucose syrup as a humectant created a more stable gluten-free dough system with improved loaf volume, reduced baking loss, and enhanced crumb moisture, which is consistent with previous studies on gluten-free bakery products [7].

The external appearance of the bread samples is presented in Table 3. The photographs demonstrate visible differences in the color and surface appearance of the samples with increasing levels of corn zein. The BZ0 sample showed the characteristic appearance of the control bread, whereas the incorporation of zein resulted in noticeable changes in the visual appearance of the bread samples.

The lowest hardness was characteristic of 6.9 for BZ20, which is 30% lower than the control. Elasticity and cohesiveness in this sample were also the highest, 0.85 and 0.57, respectively, indicating better spatial organization of the matrix. These results are consistent with data on porosity and specific volume. Sample BZ30 was characterized by increased hardness 8.3, probably due to excessive destabilization of the aqueous phase at high zein content. Thus, the addition of 10–20% zein leads to the formation of the softest and most elastic crumb.

The addition of zein affected the color characteristics of both the crumb and crust (Table 3). The L value of the crumb increased from 72.3 in BZ0 to 75.1 and 76.0 in BZ10 and BZ20, respectively, indicating a gradual lightening of the crumb. A similar tendency was observed for the crust, although the L values varied among the formulations. The increase in L* may be associated with changes in browning reactions and the dilution of buckwheat-derived pigments and phenolic compounds following the incorporation of zein. The changes in a* and b* values further indicate that zein incorporation modified the red–green and yellow–blue components of the bread color.

### 3.5. In Vitro Starch Digestion and Conditional Glycemic Index

The incorporation of corn zein significantly affected the starch digestibility profile and predicted glycemic index (pGI) of gluten-free buckwheat bread (Table 4). Rapidly digestible starch (RDS) decreased significantly (*p* < 0.05) from 48.1% in the control to 41.6% in BZ20, while BZ30 exhibited an intermediate value that did not differ significantly from BZ10 or BZ20. Conversely, slowly digestible starch (SDS) and resistant starch (RS) increased, reaching maximum values of 26.3% and 10.4%, respectively, in BZ20. A similar trend was observed for pGI, which decreased from 72.3 in the control to 67.1 in BZ20, whereas no significant differences were found between BZ0 and BZ10 or between BZ20 and BZ30. These results indicate that incorporating 20% zein most effectively reduced starch digestibility and improved the nutritional quality of the gluten-free bread.

The observed changes are attributed to the ability of zein to form a continuous hydrophobic protein network during dough mixing and baking, which partially encapsulates gelatinized starch granules and limits α-amylase accessibility, thereby reducing RDS while promoting SDS and RS formation. At higher zein incorporation to 30%, excessive protein aggregation likely weakened the uniformity of the starch–protein matrix, explaining the slight increase in RDS and pGI compared with BZ20. Although glucose syrup supplied readily fermentable sugars and improved dough development through enhanced yeast activity and moisture retention, its concentration remained constant in all formulations and therefore did not account for the differences among treatments. Instead, the lower starch digestibility observed in zein-containing breads demonstrates that the structural effect of zein predominated over the potential glycemic contribution of glucose syrup by reducing enzymatic starch hydrolysis. These findings are consistent with previous studies reporting that protein–starch interactions in gluten-free systems decrease starch digestibility and predicted glycemic response through the formation of compact composite matrices [25,33,34]. The observed changes in starch fractions may be attributed to the structuring effect of zein within the gluten-free bread matrix. The incorporation of zein can contribute to the formation of a protein-rich network surrounding or interacting with starch granules, which may reduce the accessibility of starch to digestive enzymes and consequently slow down starch hydrolysis. This effect is particularly relevant in gluten-free systems, where the absence of gluten may otherwise result in a less effective structural barrier to starch digestion. The increased SDS and RS fractions observed in BZ20 therefore suggest that a greater proportion of starch became less readily accessible to enzymatic hydrolysis, while the reduction in RDS indicates a slower initial rate of starch digestion. The concurrent decrease in pGI from 72.3 to 67.1 is consistent with this shift in starch digestibility characteristics. However, the magnitude of the pGI reduction should be considered moderate rather than substantial. Thus, zein incorporation should not be interpreted as transforming the bread into a low-glycemic product but rather as producing a more favorable starch digestion profile within the tested gluten-free formulation. However, because RS was determined using an in vitro digestion method, these results indicate a potential improvement in starch digestibility characteristics but cannot be directly extrapolated to the actual glycemic response in humans.

### 3.6. Sensory Evaluation in Bread

The sensory evaluation results are presented in Table 5. Among the tested formulations, BZ20 demonstrated the favorable sensory profile, receiving the highest scores for all evaluated attributes. In particular, BZ20 achieved scores of 8.2 for appearance, 8.1 for crumb color, 7.9 for aroma, 7.9 for taste, 8.2 for texture, and 8.5 for overall acceptability. Compared with the control sample (BZ0), the BZ20 formulation showed a clear improvement in sensory perception. The scores for appearance, taste, texture, and overall acceptability increased from 6.8 to 8.2, 6.5 to 7.9, 6.1 to 8.2, and 6.8 to 8.5, respectively. This improvement is consistent with the instrumental measurements, which showed that BZ20 had the highest specific volume (2.41 mL/g) and the lowest hardness (6.9 N) among the investigated formulations. The higher texture score of BZ20 may therefore be associated with its improved structural characteristics and reduced hardness. The higher appearance and crumb color scores may also reflect the more uniform and visually acceptable crumb structure obtained at this zein concentration. BZ10 also showed improved sensory characteristics compared with BZ0, with increases in appearance, crumb color, taste, texture, and overall acceptability scores. However, the sensory scores remained lower than those obtained for BZ20, indicating that 10% zein provided a positive but less pronounced effect on the overall sensory quality of the bread.

In contrast, increasing the zein content to 30% resulted in a decline in sensory scores compared with BZ20. The BZ30 sample received lower scores for appearance (7.4), aroma (7.4), taste (7.0), texture (7.0), and overall acceptability (7.1). The reduction in texture score is consistent with the greater rigidity observed at the higher zein concentration. These findings suggest that excessive zein incorporation may negatively affect the balance of structural and sensory properties of gluten-free bread.

Overall, the sensory results provide complementary evidence supporting the selection of BZ20 as the most favorable formulation. The 20% zein level provided the best balance of appearance, crumb color, aroma, taste, texture, and overall acceptability and was also associated with the most favorable physicochemical and textural characteristics. Thus, the sensory evaluation strengthens the practical relevance of the BZ20 formulation and addresses the potential concern that the characteristic taste and color of zein could negatively affect the acceptability of the final product.

## 4. Conclusions

The incorporation of corn zein significantly improved the technological, structural, and nutritional properties of gluten-free buckwheat bread. Among the tested formulations, bread containing 20% corn zein demonstrated the optimum performance, exhibiting the longest dough development time of 3.58 min, the highest protein strength with C2 and C3 values of 1.09 and 2.08 Nm, respectively, a peak viscosity of 1779 cP, and water absorption of 59.2%. These structural improvements resulted in superior bread quality, characterized by the highest specific volume of 2.41 mL/g, the lowest baking loss of 10.1%, and the highest crumb moisture of 41.1%. SEM analysis confirmed the formation of a dense and continuous protein–starch matrix, while starch digestibility analysis showed that rapidly digestible starch decreased from 48.1% to 41.6%, the predicted glycemic index decreased from 72.3 to 67.1, slowly digestible starch increased from 22.4% to 26.3%, and resistant starch increased from 7.8% to 10.4%. Although glucose syrup enhanced yeast activity, moisture retention, and dough development, the structural effect of zein predominated by limiting enzymatic accessibility to gelatinized starch. Sensory evaluation further confirmed the suitability of the 20% zein formulation (BZ20), which demonstrated the most favorable sensory profile, with the highest scores for appearance, crumb color, aroma, taste, texture, and overall acceptability among the tested formulations.

Overall, the results demonstrate that the incorporation of 20% corn zein is the optimal strategy for improving the rheological properties, bread quality, microstructure, and nutritional functionality of gluten-free buckwheat bread, providing an effective approach for developing gluten-free bakery products with enhanced technological performance and a lower predicted glycemic response.

## 5. Patents

Akshorayeva Gaukhar Dyusengalievna, Kakimov Mukhtarbek Mukanovich and Bolskhan Baizhigit. Method for producing gluten-free bread using corn protein. The utility model was granted patent No. 12388, issued on 12 June 2026.

## Figures and Tables

**Table 1:** Physicochemical properties of buckwheat flour and corn zein (% in product (g/100 g)).

Name of Indicator	Units (%)	Buckwheat Flour	Corn Zein
Moisture	%	12.8 ± 0.05	8.00 ± 0.023
Fat	%	2.23 ± 0.03	1.49 ± 0.045
Protein	%	11.28 ± 0.01	89.56 ± 0.023
Ash	%	2.67 ± 0.03	0.95 ± 0.065
Carbohydrate	%	71.02 ± 0.03	-

**Table 2:** Pasting and thermomechanical characteristics of buckwheat flour and zein doughs.

	Units	Samples			
		BZ0	BZ10	BZ20	BZ30
	Pasting characteristics				
Peak viscosity	cP	1105 ± 0.6 ^d^	1173 ± 0.6 ^c^	1779 ± 1.0 ^b^	2017 ± 1.0 ^a^
Pasting temperature	cP	80.5 ± 0.1 ^c^	88.7 ± 0.1 ^a^	84.05 ± 0.3 ^b^	72.6 ± 0.3 ^d^
Trough viscosity	cP	958 ± 2.5 ^c^	1129 ± 2.5 ^b^	887 ± 2.1 ^d^	1939 ± 2.0 ^a^
Final viscosity	cP	2087 ± 1.0 ^b^	1908 ± 1.7 ^c^	1615 ± 1.5 ^d^	2781 ± 1.0 ^a^
	Thermal characteristics				
Water absorption	%	55.6 ± 0.61 ^b^	58.7 ± 0.57 ^a^	59.2 ± 0.61 ^a^	59.7 ± 0.57 ^a^
Dough formation time	min	1.32 ± 0.14 ^c^	1.92 ± 0.08 ^b^	3.58 ± 0.14 ^a^	1.08 ± 0.06 ^d^
Stability time	min	9.80 ± 0.50 ^a^	8.20 ± 0.70 ^b^	9.86 ± 0.40 ^a^	8.96 ± 0.70 ^ab^
C2	Nm	0.440 ± 0.01 ^c^	0.923 ± 0.02 ^b^	1.090 ± 0.02 ^a^	0.916 ± 0.02 ^b^
C3	Nm	1.160 ± 0.05 ^d^	1.906 ± 0.01 ^b^	2.080 ± 0.03 ^a^	1.786 ± 0.04 ^c^
C5−C4	Nm	0.300 ± 0.02 ^c^	2.860 ± 0.02 ^b^	2.916 ± 0.02 ^a^	2.880 ± 0.02 ^ab^

Statistical analysis was performed using one way ANOVA (*p* < 0.05), with significant differences indicated by letters in the same column (a, b, c, d).

**Table 3:** Quality characteristics and external appearance of bread samples with different zein concentrations.

Indicator Names	Units	Samples			
		BZ0	BZ10	BZ20	BZ30
		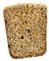	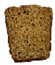	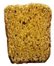	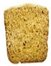
Specific volume	mL/g	2.05 ± 0.04 ^c^	2.32 ± 0.05 ^b^	2.41 ± 0.06 ^b^	2.28 ± 0.05 ^b^
Loss during baking	%	11.8 ± 0.4 ^a^	10.6 ± 0.3 ^bc^	10.1 ± 0.2 ^c^	11.2 ± 0.3 ^ab^
Crumb moisture	%	38.4 ± 0.6 ^b^	40.3 ± 0.7 ^a^	41.1 ± 0.8 ^a^	39.6 ± 0.7 ^ab^
		Color characteristics			
Crumb					
L*		72.3 ± 0.94 ^b^	75.1 ± 0.97 ^a^	76.0 ± 0.98 ^a^	74.2 ± 0.96 ^ab^
a*		2.4 ± 0.31 ^a^	2.3 ± 0.29 ^a^	2.2 ± 0.28 ^a^	2.4 ± 0.31 ^a^
b*		12.1 ± 1.57 ^a^	13.4 ± 1.74 ^a^	14.3 ± 1.85 ^a^	13.5 ± 1.75 ^a^
Crust					
L*		71.62 ± 0.93 ^c^	78.10 ± 0.15 ^a^	74.27 ± 0.96 ^b^	79.82 ± 1.03 ^a^
a*		1.58 ± 0.20 ^a^	1.42 ± 0.18 ^a^	3.24 ± 0.42 ^a^	2.39 ± 0.31 ^b^
b*		32.40 ± 4.02 ^a^	15.24 ± 1.98 ^bc^	21.68 ± 2.81 ^b^	10.99 ± 1.42 ^c^
		Texture characteristics			
Chewiness	N	3.81± 0.19 ^b^	3.43± 0.17 ^b^	3.12± 0.02 ^b^	3.56± 0.18 ^b^
Cohesiveness	dimensionless	0.51± 0.02 ^c^	0.55± 0.03 ^c^	0.57± 0.03 ^c^	0.54± 0.03 ^c^
Elasticity	dimensionless	0.78 ± 0.04 ^c^	0.82 ± 0.04 ^c^	0.85 ± 0.04 ^c^	0.80 ± 0.04 ^c^
Hardness	N	9.8 ± 0.5 ^a^	7.6 ± 0.4 ^a^	6.9 ± 0.3 ^a^	8.3 ± 0.4 ^a^

Note: Photographs show the appearance of the bread samples: BZ0 (0% zein), BZ10 (10% zein), BZ20 (20% zein), and BZ30 (30% zein). Statistical analysis was performed using one way ANOVA (*p* < 0.05), with significant differences indicated by letters in the same column (a, b, c).

**Table 4:** Starch fractions and pGI.

Samples	Units	BZ0	BZ10	BZ20	BZ30
RDS	%	48.1 ± 1.07 ^a^	44.3 ± 0.98 ^b^	41.6 ± 0.93 ^c^	42.8 ± 0.95 ^bc^
SDS	%	22.4 ± 0.27 ^c^	24.8 ± 0.29 ^b^	26.3 ± 0.32 ^a^	24.9 ± 0.29 ^b^
RS	%	7.8 ± 0.17 ^d^	9.1 ± 0.20 ^c^	10.4 ± 0.23 ^b^	9.5 ± 0.21 ^c^
pGI		72.3 ± 1.45 ^a^	69.4 ± 1.38 ^a^	67.1 ± 1.34 ^b^	68.2 ± 1.36 ^b^

Statistical analysis was performed using one way ANOVA (*p* < 0.05), with significant differences indicated by letters in the same column (a, b, c, d).

**Table 5:** Sensory evaluation scores of gluten-free bread samples using a 9-point scale.

Indicator	BZ0	BZ10	BZ20	BZ30
Appearance	6.8 ± 0.35	7.6 ± 0.52	8.2 ± 0.46	7.4 ± 0.52
Crumb color	6.8 ± 0.35	7.9 ± 0.35	8.1 ± 0.35	7.6 ± 0.52
Aroma	7.1 ± 0.35	7.5 ± 0.53	7.9 ± 0.35	7.4 ± 0.52
Taste	6.5 ± 0.53	7.2 ± 0.46	7.9 ± 0.35	7.0 ± 0.00
Texture	6.1 ± 0.35	7.4 ± 0.52	8.2 ± 0.46	7.0 ± 0.00
Overall acceptability	6.8 ± 0.35	7.6 ± 0.52	8.5 ± 0.53	7.1 ± 0.35

## Data Availability

The data are not publicly available due to research project restrictions but are available from the corresponding author upon reasonable request.

## Abbreviations

BZ0	Gluten-free bread containing 0% corn zein (control sample)
BZ10	Gluten-free bread containing 10% corn zein
BZ20	Gluten-free bread containing 20% corn zein
BZ30	Gluten-free bread containing 30% corn zein
RDS	Rapidly Digestible Starch
SDS	Slowly Digestible Starch
RS	Resistant Starch
pGI	Predicted Glycemic Index
